# Bloch points in nanostrips

**DOI:** 10.1038/s41598-023-33998-z

**Published:** 2023-04-27

**Authors:** Martin Lang, Marijan Beg, Ondrej Hovorka, Hans Fangohr

**Affiliations:** 1 Faculty of Engineering and Physical Sciences, University of Southampton, Southampton, SO17 1BJ UK; 2 Max Planck Institute for the Structure and Dynamics of Matter, Luruper Chaussee 149, 22761 Hamburg, Germany; 3 Department of Earth Science and Engineering, Imperial College London, London, SW7 2AZ UK; 4 Center for Free-Electron Laser Science, Luruper Chaussee 149, 22761 Hamburg, Germany

**Keywords:** Magnetic properties and materials, Magnetic properties and materials, Computational science

## Abstract

Complex magnetic materials hosting topologically non-trivial particle-like objects such as skyrmions are under intensive research and could fundamentally change the way we store and process data. One important class of materials are helimagnetic materials with Dzyaloshinskii-Moriya interaction. Recently, it was demonstrated that thin nanodisks consisting of two layers with opposite chirality can host a single stable Bloch point of two different types at the interface between the layers. Using micromagnetic simulations we show that FeGe nanostrips consisting of two layers with opposite chirality can host multiple coexisting Bloch points in an arbitrary combination of the two different types. We show that the number of Bloch points that can simultaneously coexist depends on the strip geometry and the type of the individual Bloch points. Our simulation results allow us to predict strip geometries suitable for an arbitrary number of Bloch points. We show an example of an 80-Bloch-point configuration verifying the prediction.

Magnetic quasiparticles with non-trivial topology^1^, such as vortices and skyrmions, are of great fundamental interest and could play an important role in novel technological applications^2^. One of the quasiparticles is the Bloch point: a single point of vanishing magnetisation^3,4^. Bloch points in single-layer materials have been studied in detail using micromagnetic^5^ and atomistic simulations^6^ and play an important role as dynamical objects in vortex-antivortex annihilation^7^ and vortex core reversal^8,9^. Their static structure has recently been imaged experimentally using X-ray vector nanotomography^10^, their dynamics was measured using transmission X-ray microscopy^11^. Long magnetic cylinders can host a stable Bloch point in the core of a vortex domain wall^12^. In this configuration the Bloch point is stabilised by the demagnetisation field if the cylinder is sufficiently large. A stable and manipulable Bloch point of two different types was also predicted in thin helimagnetic two-layer nanodisks^13^ where the two layers have opposite chirality, and the Bloch point nucleates at the interface between the layers. The demagnetisation field in these systems would not be strong enough to stabilise the Bloch point configuration. Instead, the Bloch point is stabilised by the different chiralities of the two layers. The Bloch point observed in Ref.^13^ is structurally equivalent to the meta-stable Bloch point studied in Ref.^5^. The important difference between a single-layer system and the two-layer system is that the latter stabilises the Bloch-point configuration over the vortex configuration in thin samples.

In this work, we demonstrate that two-layer nanostrips can host multiple Bloch points in an arbitrary combination of different Bloch-point types. Using finite-difference micromagnetic simulations, we explore the parameter space of strip geometry to understand for which geometry constraints such magnetisation field configurations can be realised. The Bloch point as a (meta)-stable topological excitation and quasiparticle opens up new avenues of fundamental research. In particular, we can now start to investigate individual and collective behaviour of Bloch points such as the melting transition of a “crystal” of Bloch points similar to vortices in high-temperature superconductors^14^ or skyrmion lattices^15,16^. We conclude our study with a demonstration of encoding a 10-byte string using 80 Bloch points: we identify one Bloch-point type with the binary “1” and the other type with the binary “0” to encode and store the equivalent of an 80-bit long sequence.

The concept of a Bloch point in a two-layer system^13^ is explained in Fig. 1, where we start from vortex configurations in single-layer materials, and then stack them on top of each other to obtain the Bloch-point configuration of the magnetisation field. Figure 1a shows schematically the four possible vortex configurations we can encounter in a thin layer of ferromagnetic material due to the competition between ferromagnetic exchange and demagnetisation energy. The vortex core (polarisation *P*), pointing in the out-of-plane (*z*) direction, can either be pointing to $+z$ ($P=+1$) or $-z$ ($P=-1$) direction, and—independently—the circularity *c* of the magnetisation around the vortex core can either be clock-wise ($c=-1$) or counter-clock-wise ($c=+1$)^17^. The product of polarisation and circularity can be defined as the chirality of the vortex and describes the relative orientation of the moments in the vortex (i.e. the handedness of the vortex). By adding the Dzyaloshinskii-Moriya (DM) interaction to the system, the preferred combination of polarisation and circularity is determined by the chirality of the material (not to be confused with the chirality of the vortex), *i.e.* the sign of the DM interaction strength *D*. For a given nonzero *D* two of the four vortex realisations are energetically favourable: vortices in a chiral material have a lower energy if their vortex chirality (handedness) matches the chirality of the material.

Figures 1b and c show how a Bloch-point configuration can be realised by stacking vortex configurations with the same circularity and opposite polarisation on top of each other. The Bloch point emerges at the interface between the two vortex cores of opposite polarisation and the top- and bottom-layer materials must have opposite signs of *D* to stabilise the Bloch-point configuration. Despite the high exchange energy density at the Bloch point, the configuration is stabilised by the exchange coupling across the comparatively large interface area between the layers that ensures the same circularity in both layers. Two different types of Bloch points can be realised with the magnetisation vectors of the vortex cores pointing either inwards (head-to-head Bloch point (HH), Fig. 1b) or outwards (tail-to-tail Bloch point (TT), Fig. 1c). Other combinations of vortices in the two layers either have a higher energy or are not stable: when we stack two vortices with the same circularity and polarisation (in a handedness that matches the material’s chirality in the bottom layer) we find a vortex extending throughout the two layers (and shrinking in diameter in the top layer) that is higher in energy because the vortex handedness does not match the material’s chirality in the top layer (resulting in a large DMI energy). If we stack two vortices with the same polarisation but opposite circularity the handedness of the vortices matches the chirality of the material in both layers. However, this stacking results in a large interface with magnetic moments pointing in opposite direction (due to the opposite circularity) which has a large exchange energy. This configuration could not be stabilised and instead converts to the Bloch-point configuration with vortices having the same circularity but opposite polarisation.

Figure 1d shows the magnetisation vector field for a single head-to-head Bloch point from our finite-difference micromagnetic simulations, and the colour represents the *z* component of the magnetisation. Figure 1e shows the corresponding plot for a tail-to-tail Bloch point. In the simulation, the Bloch point forms at the centre of eight discretisation cells as shown in the insets of Fig. 1d and e where the magnetisation of the discretisation cells is visualised by the cones. The Bloch point is located at the intersection of the three isosurfaces $m_{x, y, z} = 0$ where the magnetisation vanishes. The tail-to-tail Bloch point is similar to the circulating Bloch point in the nomenclature of Malozemoff and Slonczewski [Figure 9.1,^18^], however the rotation of the magnetic moments in the interface plane deviates from the perfect $90^{\circ }$ rotation around the *z* axis. For the head-to-head Bloch point no direct equivalent is shown in Ref.^18^. Using the nomenclature of Malozemoff and Slonczewski to describe both configurations, one could call the tail-to-tail Bloch point a “divergent circulating” Bloch point and the head-to-head Bloch point a “convergent circulating” Bloch point. Other literature refers to such configurations as spiraling Bloch points^11^ or vortex Bloch points^19^. To better show the magnetisation around the Bloch point we show the eight magnetisation discretisation cells surrounding the Bloch point in Fig. 1f and g for the HH and TT configuration, respectively. We interpolate the magnetisation in the eight cells to obtain the magnetisation direction on the cell corners (using VTK^20^). We highlight the Bloch point position with a small green sphere to guide the eye. Figure 1h and i show a reduced number of magnetisation directions around the Bloch-point positions. Here, we see that the magnetic moments above and below the Bloch point point towards the Bloch point in the HH configuration and away from the Bloch point in the TT configuration. Furthermore, we can see the in-plane circularity at the interface. Figure 1h and i show that the numerically obtained configurations closely match the circulating Bloch point described in the book by Malozemoff and Slonczewski^18^.

**Figure 1:**
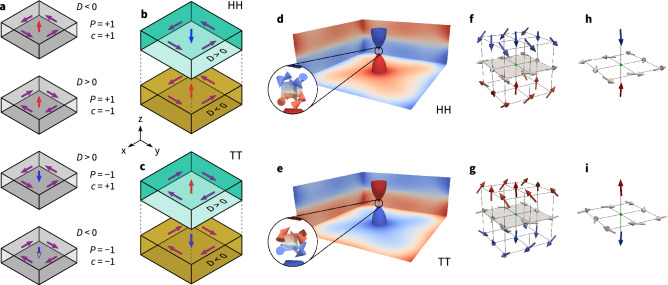
(**a**) In a single layer of magnetic material four different vortices, with polarisation $P=\pm 1$ and circularity $c=\pm 1$, can form as a consequence of the competition between exchange energy and demagnetisation. Adding DMI couples circularity and polarisation. (**b**, **c**) By stacking two layers with opposite sign of the DM energy constant *D*, a Bloch point can be stabilised. The Bloch point can be of type head-to-head (**b**) or tail-to-tail (**c**). In the figure, the two layers are, for better clarity, separated in *z* direction as indicated by the grey dashed lines. (**d**, **e**) Simulation result for a single head-to-head (**d**) and tail-to-tail (**e**) Bloch point. The isosurfaces (of paraboloidal-like shape) near the centre show $m_{z}=\pm 0.9$, colour indicates the *z* component. They are convenient to locate the Bloch point that is situated between them. The insets show three isosurfaces for $m_{x} = 0$, $m_{y} = 0$, and $m_{z} = 0$, respectively. The Bloch point is located at the intersection of the three isosurfaces where the magnetisation vanishes. The cones in the insets indicate the magnetisation directions in the eight discretisation cells surrounding the Bloch point. The magnetisation around the Bloch point is show in more details in (**f**, **h**) for the HH configuration and in (**g**, **i**) for the TT configuration. See the main text for additional details.

The paper is organised as follows. First, we discuss two different configurations containing two Bloch points each in the "Two Bloch points" section. We distinguish between configurations of two same-type Bloch points (e.g. two HH Bloch points) and two opposite-type Bloch points (e.g. one HH and one TT Bloch point). These two pairs of Bloch points exhibit all features of a configuration containing multiple Bloch points. In the "Parameter-space diagram and energy density" section, we demonstrate that multiple Bloch points can coexist in rectangular two-layer nanostrips using micromagnetic simulations. We find that all possible sequences of head-to-head and tail-to-tail Bloch points can be realised. We find that micromagnetic configurations that contain different combinations of Bloch points have different energy densities depending on the number of neighbouring Bloch points of the same type. We focus on systems containing up to eight Bloch points initially. Based on our results, we can predict a suitable strip geometry for an arbitrary number of Bloch points. To verify this prediction we present one example configuration containing 80 Bloch points in the "Predicting strip geometries for larger systems" section.

## Results

### Two Bloch points

**Figure 2:**
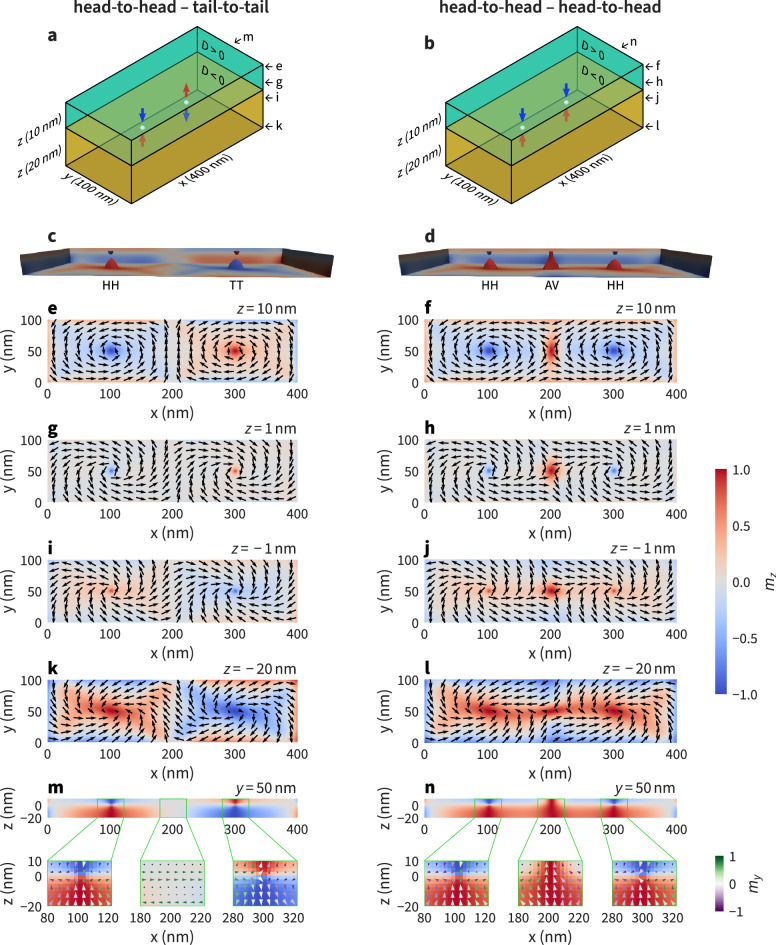
Magnetisation profile of the two fundamentally different configurations containing two Bloch points: opposite-type Bloch points (head-to-head and tail-to-tail, left column) and same-type Bloch points (head-to-head and head-to-head, right column). The 3D renderings in (**c**) and (**d**) show isosurfaces for $m_{z} = \pm 0.9$, colour indicates the *z* component. Several different cut planes in *xy* and *xz* are shown to reveal the three-dimensional structure of the Bloch points forming at the interface ($z=0\,\textrm{nm}$). For the *xz* plane (subfigures **m** and **n**) we also show enlarged plots around the Bloch point and antivortex position. The cones in (**m**) and (**n**) are coloured according to their $m_y$ component, as indicated by the small colour bar in the right bottom corner of the figure.

A pair of neighbouring Bloch points in multi-Bloch-point configurations can occur in two fundamentally different combinations. The Bloch points can either be of the same type, for example: a head-to-head (HH) Bloch point next to another HH Bloch point (HH-HH) as shown in the right column of Fig. 2. Alternatively, the Bloch points can be of opposite type, for example a HH Bloch point next to a tail-to-tail (TT) Bloch point (HH-TT) as shown in the left column of Fig. 2.

The topmost row (Fig. 2a, b) shows a schematic drawing of the nanostrip highlighting the two-layer structure and the geometry. Additionally, position and type of the Bloch points visible in the simulations are indicated with arrows, where the colour of the arrows encodes the *z* component of the magnetisation (red: $+z$, blue: $-z$).

Figure 2c and d show 3D renderings of the simulation results. The isosurfaces (as used by Hertel and Schneider^7^ to visualise magnetic vortices) show $m_{z}=\pm 0.9$ ($\textbf{m}$ is the normalised magnetisation), colour indicates the *z* component. The isosurfaces above/below the Bloch point have a paraboloidal-like shape pointing towards the Bloch point (similar to the single-Bloch-point simulation results in Fig. 1c, d). The Bloch point itself is not directly visible in this visualisation. The configuration in Fig. 2d additionally contains one antivortex between the two Bloch points. The $m_{z} = 0.9$ isosurface of the antivortex extends throughout the whole thickness (*z* direction) of the two-layer system. The antivortex core shrinks towards the top sample boundary.

To reveal the full three-dimensional structure of the magnetisation field surrounding the Bloch points the magnetisation of each configuration is plotted in five different cut planes for each column (as indicated in the schematic drawings Fig. 2a, b). Four different cut planes show the magnetisation in the *xy* plane (Fig. 2e – l), at the top sample boundary ($z=10\,\textrm{nm}$) in Fig. 2e and f, above the interface ($z=1\,\textrm{nm}$) in Fig. 2g and h, below the interface ($z=-1\,\textrm{nm}$) in Fig. 2i and j, and at the bottom sample boundary ($z=-20\,\textrm{nm}$) in Fig. 2k and l. Colour encodes the *z* component of the magnetisation vector field, arrows the in-plane component.

Figure 2m and n show the *z* component of the magnetisation in an *xz* cut plane going through the Bloch points at $y=50\,\textrm{nm}$. Magnified subplots show the full magnetisation around the Bloch-point positions and in the centre region between the two Bloch points. The colour of the cones in the magnified areas encodes the *y* component of the magnetisation vector field.

The results shown in Fig. 2 show that Bloch points form at $x\approx 100\,\textrm{nm}$ and $x\approx 300\,\textrm{nm}$ in both cases, *i.e.* in both columns. Bloch-point pairs of the opposite type (Fig. 2, left column) show opposite circularity of the magnetisation within the *xy* plane around the Bloch-point cores. In this case, the in-plane magnetisation (*xy* component) between the two Bloch points (from $x\approx 100\,\textrm{nm}$ to $x\approx 300\,\textrm{nm}$) shows a smooth transition from one Bloch point to the other. Focusing only on the top layer (or focusing only on the bottom layer), the configuration can also be described as a micromagnetic configuration containing two vortices of opposite polarisation and circularity (most clearly seen in Fig. 2e). In contrast, an additional antivortex forms between two same-type Bloch points (Fig. 2, right column) at $x\approx 200\,\textrm{nm}$ to mediate between the incompatible magnetisation configurations that originate from the two same-circularity vortices. Differing from the magnetisation of the Bloch-point cores, the magnetisation of the antivortex core (at $x \approx 200\,\textrm{nm}$) does not change significantly along the *z* direction (middle inset in Fig. 2n). This configuration can be described as a micromagnetic configuration containing a cross-tie domain wall^21,22^ in each layer. The cross-tie wall consists of alternating vortices and antivortices, the structure is most clearly visible in Fig. 2f.

### Parameter-space diagram and energy density

The spatially averaged energy density (total energy of the system divided by the system volume) of a Bloch-point configuration depends on the number of Bloch points, their individual types, and the strip geometry. Furthermore, different spatial arrangements can be realised, *e.g.* four Bloch points on a line, or in the corners of a rectangle or diamond shape. Here, we only consider magnetisation configurations containing between one and eight Bloch points in a row, distributed in *x* direction (strip length) and centred in *y* direction (strip width).

In Fig. 2 we have seen the two fundamentally different configurations containing two Bloch points, head-to-head and tail-to-tail (HH-TT), and head-to-head and head-to-head (HH-HH). Now we investigate a system containing three Bloch points. In total, eight configurations can be realised. Three configurations are fundamentally different, namely HH-HH-HH, HH-HH-TT, and HH-TT-HH, because they contain distinct numbers of additional antivortices. The other five configurations are equivalent either because HH and TT swap roles (*e.g.* TT-TT-TT), or because of the symmetry of the system geometry along the *x* axis (*e.g.* TT-HH-HH).

**Figure 3:**
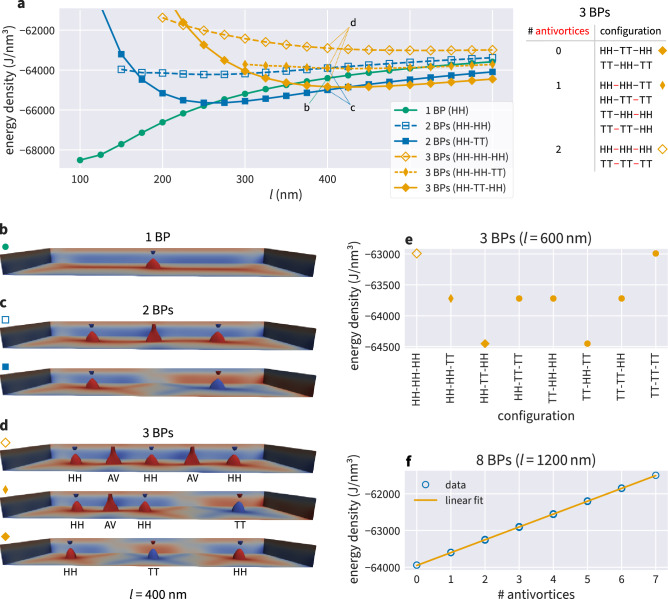
(**a**) Energy densities for energetically different micromagnetic configurations containing at most three Bloch points for different strip lengths *l* at a strip width $w=100\,\textrm{nm}$. Simulations have been performed in steps of $\delta l = 25\,\textrm{nm}$, the solid lines are shown to guide the eye. (**b**–**d**) Magnetisation profiles for the six different configurations shown in (**a**) at $l=400\,\textrm{nm}$. Isosurfaces show $m_{z}=\pm 0.9$, colour indicates the z-component. (**e**) Energy density for all possible configurations containing three Bloch points. The first three configurations are show in (**d**) as indicated with the distinct marker symbols (at a different strip length). The table in (**a**) lists all different configuration containing three Bloch points, highlighting the number and position of the additional antivortices in the different configurations. (**f**) Energy densities for all configurations containing eight Bloch points. Energy densities for a fixed number of antivortices are nearly identical but cause some “smearing” of the marker symbols.

The fundamentally different configurations (HH-HH-HH, HH-HH-TT, HH-TT-HH) are shown in Fig. 3d in a system with strip length $l=400\,\textrm{nm}$. We find one and two additional antivortices (AVs) for configurations HH-HH-TT and HH-HH-HH, respectively. The table in Fig. 3 lists all eight configurations and the respective number of antivortices. In different terms, the three configurations in Fig. 3d could also be described as (in each layer) containing one long cross-tie wall (top), a cross-tie wall and one vortex (middle), and three vortices (bottom).

For each of the three fundamentally different configurations, we compute the spatially averaged energy density of the micromagnetic configuration and plot the three values in Fig. 3a (at $l=400\,\textrm{nm}$).

We find that the micromagnetic configuration containing three Bloch points in the HH-TT-HH configuration has the lowest energy density and the micromagnetic configuration containing three Bloch points in the HH-HH-HH configuration the highest energy density. Hereinafter, we will refer to a micromagnetic configuration containing *n* Bloch points as the *n*-Bloch-point configuration to simplify the text. Note that we always mean the entire micromagnetic configuration with embedded Bloch points and not isolated Bloch points, e.g. when talking about the “energy of the Bloch points” (which should be read as “energy of the micromagnetic configuration containing Bloch points”). It is well-known that the Bloch points “them-self”, i.e. the point singularities, do not affect the energy of the micromagnetic configuration^3^. We will later discuss in more detail that the presence of antivortices between Bloch points generally increases the energy density of the system. The alternating configuration HH-TT-HH does not contain any antivortices, as these are only needed to mediate the local rotation of the magnetisation between neighbouring vortices that enclose Bloch points of the same type.

The three yellow lines (filled and open diamonds) in Fig. 3a show the spatially averaged energy density for the three different configurations as a function of strip length. Not all configurations are stable for all strip lengths: for example the HH-HH-TT configuration is only stable for $l\ge 300\,\textrm{nm}$. If we try to create the three-Bloch-point configuration HH-HH-TT in a shorter nanostrip, *e.g.* at $l=275\,\textrm{nm}$, then the configuration is not stable and will change into a lower-energy configuration, in this case the HH-TT configuration containing only two Bloch points. In other words, the cross-tie walls enclosing the HH-HH Bloch-point pair are not stable in too short nanostrips and collapse. This has in detail been studied for cross-tie walls in single-layer materials^7^. We can see that the energy generally increases with increasing number of antivortices as mentioned in the previous paragraph. However, there is a deviation visible for $l\le 225\,\textrm{nm}$ where the HH-HH-HH configuration has a lower energy density than the HH-TT-HH configuration. This deviation is a result of the short strip length near the stability limit. We exclude these regions near the minimal stability strip length in the rest of our discussion.

The blue filled and open squares in Fig. 3a show the energy density for a system containing only two Bloch points. The corresponding magnetisation field for $l=400\,\textrm{nm}$ is visualised in Fig. 3c, and in more detail in Fig. 2. The green circles in Fig. 3a show the energy density for a configuration containing a single Bloch point, and its magnetisation configuration for $l=400\,\textrm{nm}$ is shown in Fig. 3b.

For a given strip length *l* we describe the configuration with the lowest energy density as the energetically most favourable configuration: below $l=250\,\,\textrm{nm}$ a single Bloch point (green circles) has the lowest energy density. (Note that the energy of the micromagnetic configuration containing one Bloch point is lower than the energy of a vortex expanding throughout the system because of the two-layer system with opposite chirality in the two layers.) Two opposite-type Bloch points (blue squares) have the lowest energy density for $250\,\,\textrm{nm}< l \le 400\,\,\textrm{nm}$ and three Bloch points of alternating opposite type (yellow diamonds) have the lower energy density above $l=400\,\,\textrm{nm}$.

Figure 3e shows a representation of the energy densities for all possible three-Bloch-point configurations at $l=600\,\textrm{nm}$. As already discussed, there are three fundamentally different configurations characterised by the number of additional antivortices contained in the configuration (as shown in the table in Fig. 3). Different realisations of the same configuration type (swapping HH and TT or using the strip symmetry) exhibit the same energy density.

**Figure 4:**
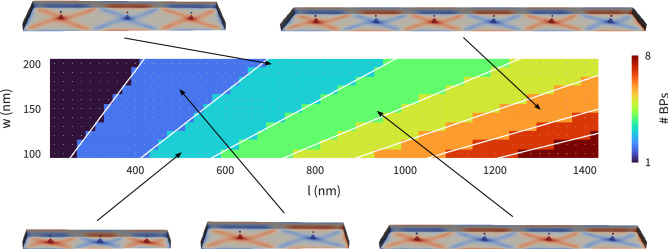
Parameter-space diagram showing the energetically most favourable Bloch point number as a function of length *l* and width *w*. All configurations contain Bloch points of alternating opposite type (so the micromagnetic configurations do not contain additional antivortices). Magnetisation profiles for selected configurations reveal the similarity of the different configurations, isosurfaces show $m_{z}=\pm 0.9$.

In Fig. 3a we have seen that the number of Bloch points in the energetically most favourable configuration changes depending on the strip length *l*. Figure 4 contains a parameter-space diagram showing the energetically most favourable configuration as a function of the strip length and strip width, using the Bloch point number as a label. To create Fig. 4, we ask for each strip length *l* and a given strip width *w*, which configuration has the lowest energy density. For example: all data points in Fig. 3a are for a width of $w=100\,\textrm{nm}$. Close to $l \approx 400\,\textrm{nm}$, we see that for $l \le 400\,\textrm{nm}$ the two-Bloch-point configuration HH-TT (blue squares) has the lowest energy density but that for $l>400\,\textrm{nm}$ the three-Bloch-point configuration HH-TT-HH (yellow diamonds) has the lowest value. Figure 4 shows (for $w=100\,\textrm{nm}$ on the *y* axis) that the two-Bloch-point configuration has the lowest energy density up to $l \approx 400\,\textrm{nm}$, and the three-Bloch-point configuration for larger *l* (up to $l \approx 600\,\textrm{nm}$). All configurations with lowest energy density are of the alternating Bloch-point type, *i.e.* left and right neighbours of a HH Bloch point are always of type TT, and vice versa (see discussion below), and hence do not contain additional antivortices.

Figure 4 shows that with increasing strip length, the number of Bloch points that are present in the lowest-energy-density configuration (as shown Fig. 3a for $l \le 600\,\textrm{nm}$) increases: for nanostrips with lengths above $l \approx 1300\,\textrm{nm}$ and width $w=100\,\textrm{nm}$, we find eight Bloch points. Furthermore, Fig. 4 shows that increasing the width of the nanostrip leads to a reduced number of Bloch points in the energetically most favourable configuration.

Figure 4 also shows magnetisation profiles for selected configurations revealing the similarities in the magnetisation profile in different strip geometries. The isosurfaces show $m_{z} = \pm 0.9$, colour indicates the *z* component. All configurations shown in Fig. 4 contain Bloch points of alternating opposite type, *i.e.* all the lowest-energy configurations do not contain antivortices.

In the discussion of the fundamentally different configurations containing three Bloch points, we have noted that the different configurations can be characterised by the number of additional antivortices contained in the structure. Figure 3f summarises similar findings for eight Bloch points where configurations can contain between zero antivortices (Bloch points of alternating opposite type) and seven antivortices (all Bloch points of the same type). In total, 256 configurations can be realised. The plot in Fig. 3f shows the data for all configurations and a linear fit to the data. Different realisations with the same number of antivortices cannot be distinguished in this plot as their energies are nearly identical but cause slight vertical shifts of the blue open circles used to mark the energy densities of the different configurations for intermediate numbers of antivortices. The energy density increases linearly with the number of antivortices (or equivalently, the cross-tie wall size).

There is an important difference between three Bloch points (Fig. 3e) and eight Bloch points (Fig. 3f). For a fixed number of antivortices, all different three-Bloch-point configurations are equivalent because of the system’s symmetry (globally replacing HH with TT and vice versa (and adjusting the antivortex polarisation) or $\pi$ rotations around the *z* direction) and therefore must have the same energy density. Let us give two examples: we start from the configuration HH-HH-TT. First, we can obtain an equivalent configuration by swapping HH and TT Bloch points, namely the configuration TT-TT-HH. Globally swapping all Bloch-point types (and adjusting the antivortex polarisation) does not affect the system’s physics in the context discussed here. Second, we can rotate the system around the *z* direction and obtain the configuration TT-HH-HH. Again, the two configurations are equivalent. However, for eight Bloch points we additionally find that different configurations that are not related via symmetry (globally swapping HH and TT or rotating the system around the *z* direction) also have the same energy density if they contain the same number of antivortices. For example, the configurations HH-HH-HH-HH-HH-HH-HH-TT and HH-HH-HH-HH-TT-TT-TT-TT both contain 6 antivortices (located between neighbouring same-type Bloch points, i.e. HH-HH and TT-TT pairs) but cannot be transformed into each other by swapping HH and TT or rotating the system. Yet, they exhibit the same spatially averaged energy density. Our findings suggest that the energy density of the micromagnetic configuration around the Bloch points is independent of the configuration around other Bloch points in the system (the Bloch point itself, i.e. the point singularity, is known to not affect the energy of the micromagnetic configuration containing it^3^). The energy density of any Bloch-point configuration can be obtained from a configuration containing Bloch points of alternating opposite type with additional contributions originating from the additional antivortices between neighbouring same-type Bloch points.

This is the reason for the lowest-energy-density configurations shown in Fig. 4 consisting of pairs of Bloch points of alternating type: for same-type neighbours an antivortex is required to mediate the magnetisation between the Bloch points of the same type, and the presence of such antivortices would increase the spatially averaged energy density.

We can make one additional observation in Fig. 3a. The energy density of a configuration changes as a function of strip length *l*. All configurations containing two or three Bloch points have one energy minimum at a certain length that we call the *optimal length* $l_{\textrm{o}}$. For example, the optimal length for the HH-TT configuration (blue filled squares in Fig. 3a) is $l_{\textrm{o}} \approx 275\,\textrm{nm}$.

### Predicting strip geometries for larger systems

So far, we have focused on small systems containing at most eight Bloch points. Based on this information we can predict strip geometries suitable for an arbitrary number of Bloch points.

Figure 3a shows that meta-stable configurations containing multiple Bloch points can be realised over a broad range of strip lengths but need a certain minimal strip length. This minimal length depends on the number of Bloch points and additional antivortices in the configuration. In our simulations we also see that a certain maximum strip length cannot be exceeded for a given configuration. If the strip is too long the configuration could not be stabilised and additional Bloch points appear. Furthermore, Fig. 3a shows that all configurations have a minimum in the energy density at a certain optimal length $l_{\textrm{o}}$.

**Figure 5:**
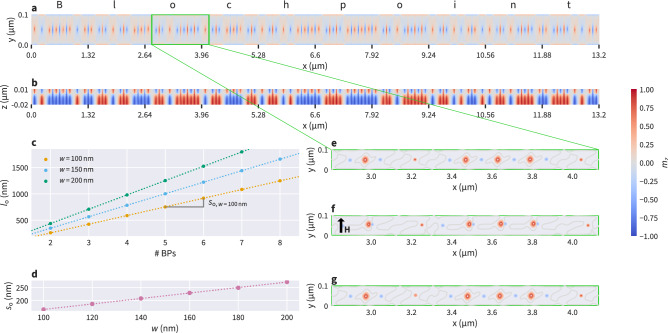
(**a**, **b**) ASCII encoding of the string Blochpoint using 80 Bloch points. Cross sections show (**a**) the *xy* plane at $z=1\,\textrm{nm}$ and (**b**) the *xz* plane at $y=50\,\textrm{nm}$. The strip length is chosen according to the predicted value for a nanostrip with width $w=100\,\textrm{nm}$. Labels on the *x* axis mark blocks of eight Bloch points, *i.e.* individual bytes. Note that the aspect ratio is not correct in order to improve visibility. (**c**) Optimal lengths $l_{\textrm{o}}$ for two to eight Bloch points. The fit is used to predict lengths for more than eight Bloch points. (**d**) Optimal Bloch point distance $s_{\textrm{o}}$ as a function of strip width *w*. (e – g) show an enlarged part of the nanostrip (correct aspect ratio) as highlighted in (**a**) to demonstrate the stability of the configuration: (**e**) initial configuration after energy minimisation; (**f**) an external magnetic field $H= 0.25\,\textrm{T} / \mu _0$ is applied in the $+y$ direction for $0.5\,\textrm{ns}$; (**g**) after removing the external field the system evolves freely and converges back to the initial state (snapshot after $5\,\textrm{ns}$). (**e**–**g**) show contour lines of the $m_z$ component to improve visibility of the disturbance introduced by the external magnetic field. Bloch points are located at the small red and blue dots, the larger red circles show the additional antivortices.

To predict strip geometries suitable for an arbitrary number of Bloch points we focus on configurations containing up to eight Bloch points of alternating opposite type. We find that the optimal length $l_{\textrm{o}}$ increases linearly with the number of Bloch points (Fig. 5c) with the slope defining the optimal Bloch point spacing $s_{\textrm{o}}$. Furthermore, we find that $s_{\textrm{o}}$ increases linearly with increasing strip width (Fig. 5d). We obtain $s_{\textrm{o}, w=100\,\textrm{nm}} \approx 165\,\textrm{nm}$ and $s_{\textrm{o}, w=200\,\textrm{nm}} \approx 272\,\textrm{nm}$ with an estimated accuracy of $\delta s_{\textrm{o}} \approx 3\,\textrm{nm}$. These observations lead to our working hypothesis that the ideal Bloch-point spacing $s_{\textrm{o}}$ is independent of the number of Bloch points in the system and suitable to predict geometries for more than eight Bloch points. This prediction can be used for arbitrary configurations not only alternating opposite-type Bloch points.

As an illustrative example, we simulate one specific configuration containing 80 Bloch points, encoding the 10-character word Blochpoint in ASCII code (eight bits per letter). We simulate a strip with the predicted length $l = 80 s_{\textrm{o}} ={13.2}\,{\upmu \hbox {m}}$ at a width of $w=100\,\textrm{nm}$ and with $s_{\textrm{o}} = 165\,\textrm{nm}$.

We minimise the energy of a suitable initial configuration resulting in the 80-bit configuration as shown in Fig. 5a, b. The cross sections show the *xy* plane at $z=1\,\textrm{nm}$ (Fig. 5a) and the *xz* plane at $y=50\,\textrm{nm}$ (Fig. 5b). Note that the aspect ratio is not correct in order to improve visibility. Figure 5e shows contour lines for $m_z$ for a part of the nanostrip (correct aspect ratio) as indicated in Fig. 5a. Bloch points in Fig. 5e are located at the small red and blue dots. The larger red circles ($m_z > 0.5$) show antivortices between same-type Bloch points.

To test the stability of the 80-Bloch-point configuration we apply a short magnetic field pulse in the $+y$ direction ($H=25\,\textrm{mT}/\mu _0$, applied for $t=0.5\,\textrm{ns}$). The modified magnetisation field configuration at the end of the $0.5\,\textrm{ns}$ period is shown in Fig. 5f. Then, we set the applied field back to zero and let the system evolve freely by carrying out a time-integration. We find that the magnetisation converges back to the initial state: Fig. 5g shows the configuration after $t=5\,\textrm{ns}$ of free relaxation.

To understand the robustness of the predicted geometry, we vary the strip length *l* and find that the desired 80-Bloch-point configuration can be stabilised over a range of strip geometries. The minimal strip length is around $0.66 l_{\textrm{o}}$ the maximal strip length around $4l_{\textrm{o}}$.

Within the range of stability of the 80-bit configuration ($0.66l_{\textrm{o}} \le l \le 4l_{\textrm{o}}$), we find that the length $l_{\textrm{o}}$ is closer to the lower stability boundary ($\approx 0.66 l_{\textrm{o}}$) than to the upper limit ($\approx 4l_{\textrm{o}}$). This is consistent with the energy density curve for the HH-TT configuration in Fig. 3a (blue filled squares) where we see that the energy density as a function of the strip length is asymmetric, and that its energy minimum, located at strip length $l_{\textrm{o}}$, is located at a comparatively small strip length within the range of possible strip lengths over which the configuration is meta-stable (stability limits are not visible in Fig. 3).

## Discussion

The Bloch-point configuration originates from vortices with identical circularity, but opposite polarisation, which are stabilised through the DMI of the material, which fixes the core orientation relative to circularity through the left- or right-handed chirality. The Bloch points form an interesting topological excitation in a helimagnetic system, which extends the set of well-known magnetic structures such as domain walls, vortices, and skyrmions. In the geometry described here, the Bloch points are in equilibrium and can be manipulated (e.g. with external magnetic fields).

We have found remarkable features of multiple interacting Bloch points in two-layer nanostrips. The two different types—head-to-head (HH) and tail-to-tail (TT)—can be geometrically arranged in any arbitrary order, and this magnetisation configuration resembles a meta-stable configuration (within certain constraints on the strip width and length). The spatially averaged energy density for a system with *n* Bloch points increases in fixed steps. The number of steps scales linearly with the number of antivortices in the configuration (or equivalently: the number of neighbouring same-type Bloch points). We can determine an optimal Bloch-point spacing $s_{\textrm{o}}$ between Bloch points within a line of Bloch points (corresponding to a distance over which a Bloch point extends).

In the following, we speculate about possible future applications of Bloch points. One key-feature distinguishing Bloch points from many other particle-like magnetic configurations is the demonstrated coexistence of Bloch points of two different types in a single sample making Bloch points an interesting candidate for binary data representation. In the racetrack-like designs^23,24^, when realised with magnetic excitations of which only one type exists—such as skyrmions—we need to ensure that skyrmions keep their relative positions to be able to interpret the presence of a skyrmion as 1 and the absence of a skyrmion as 0. The two different types of Bloch points presented here could be used to encode binary data without the need to rely on fixed spacing of magnetic objects: a HH configuration could represent “1” and a TT configuration could represent “0”. In the context of skyrmion-based realisation of the racetrack approach, other ideas to overcome the fixed-spacing requirement include the use of a combination of skyrmion tubes and chiral bobbers^25^ and the two-lane racetrack memory^26^.

On the way towards possible applications of Bloch points many more questions need to be addressed. These are related to Bloch point manipulation (movement, switching, creation/annihilation) in the two-layer system with precise control over individual Bloch points, sensing of Bloch points, and to the thermal stability of Bloch points in general and energy barriers between different configurations containing multiple Bloch points. Previous works have studied the manipulation of Bloch points in bubble memories^18^. Creation and annihilation of Bloch points as dynamic objects during vortex-antivortex annihilation has been studied in Ref.^7^. While these works demonstrate that manipulating Bloch points is possible more insights into manipulation of stable Bloch points in the two-layer system will be required.

In summary, we have demonstrated that two-layer FeGe nanostrips can host multiple Bloch points in any combination of head-to-head and tail-to-tail. Based on our simulations containing up to eight Bloch points, we can predict strip geometries suitable for an arbitrary number of Bloch points. We have verified this prediction by studying a system containing 80 Bloch points.

## Methods

### System

We simulate rectangular two-layer nanostrips with opposite chirality (opposite sign of *D*) in the two layers. We vary strip length and width, the thickness of both layers is fixed (bottom layer: $20\,\textrm{nm}$, top layer: $10\,\textrm{nm}$). We focus on up to eight Bloch points and accordingly choose nanostrips with lengths between $100\,\textrm{nm}$ and $1400\,\textrm{nm}$, and widths between $100\,\textrm{nm}$ and $200\,\textrm{nm}$. The energy equation

1 $$\begin{aligned} E = \int \textrm{d}^3r \left( w_{\textrm{ex}} + w_{\textrm{dmi}} + w_{\textrm{d}} \right) \end{aligned}$$

contains exchange energy density $w_{\textrm{ex}}$, bulk Dzyaloshinskii-Moriya energy density $w_{\textrm{dmi}}$, and demagnetisation energy density $w_{\textrm{d}}$. The magnetisation dynamics is simulated using the Landau-Lifshitz-Gilbert equation^27,28^:

2 $$\begin{aligned} \frac{\partial \textbf{m}}{\partial t} = \gamma ^* \textbf{m} \times \textbf{H}_{\textrm{eff}} + \alpha \textbf{m} \times \frac{\partial \textbf{m}}{\partial t}, \end{aligned}$$

where $\gamma ^* = \gamma (1 + \alpha ^2)$, with $\gamma$ being the gyromagnetic ratio and $\alpha$ Gilbert damping. Material parameters are based on FeGe^29^: $A = 8.87\,\textrm{pJ}\,\textrm{m}^{-1}$, $D = 1.58\,\textrm{mJ}\,\textrm{m}^{-2}$, $M_{\textrm{s}} = 384\,\textrm{kA}\,\textrm{m}^{-1}$, $\alpha =0.28$. We use finite-difference micromagnetic simulations to minimise the energy. All simulations are done using Ubermag^30–32^ with OOMMF^33^ as computational backend and an extension for DMI of crystalclass T^34,35^.

### Simulation procedure

All simulations in this study follow a three-step initialisation and minimisation scheme: (i) initialisation, (ii) fixed minimisation, (iii) free minimisation. In the micromagnetic framework the system is studied at zero temperature, *i.e.* without thermal fluctuations. Therefore, it is only possible to find local minima that are accessible from the initial configuration. Starting from experimentally feasible initial configurations, such as full saturation, we are able to find magnetisation configurations containing a single or multiple Bloch points depending on the strip geometry.

To facilitate the process of studying arbitrary Bloch-point configurations, independent of the strip geometry in a systematic way, we have developed a simulation scheme that guarantees a magnetisation configuration containing a predictable number of Bloch points. We note that this scheme can probably not be applied directly to an experimental set-up.

For the initialisation, step (i), we start by dividing the nanostrip into equally sized regions (in *x* direction), one region per Bloch point. To enforce the formation of a Bloch point, the magnetisation in each region is initialised as follows: for a head-to-head Bloch point we initialise the centre region of the topmost layer of cells with $\textbf{m} = (0, 0, -1)$ and all other cells with $\textbf{m} = (0, 0, 1)$. A region hosting a tail-to-tail Bloch point is initialised with reversed *z* component of the magnetisation (see supplementary Fig. 1 for a schematic plot of the different subregions). We then minimise the energy in two steps (supplementary Fig. 2). During the first energy minimisation, step (ii), we keep the magnetisation of the topmost cells—initialised with reversed magnetisation—and a similarly sized layer of cells at the bottom sample boundary fixed. This ensures the formation of a Bloch point at the interface between the two layers. The second energy minimisation, step (iii), is done without any fixed cells, *i.e.* magnetisation in all cells can freely change, and Bloch points could move in any direction to further minimise the energy of the configuration. In this step, the system can find the local energy minimum.

### Classification

In the micromagnetic framework, it is not possible to directly observe Bloch points because of the fixed norm of the magnetisation vector. A single Bloch point is characterised by the integral value of the topological charge density over a closed surface *A* surrounding the Bloch point^11^:

3 $$\begin{aligned} S = \frac{1}{4\pi } \int _A \textrm{d}\textbf{A} \cdot \textbf{F} = \pm 1, \end{aligned}$$

where $\textbf{F}$ is the emergent magnetic field^36,37^. The components of $\textbf{F}$ are defined as:

4 $$\begin{aligned} F_i = \textbf{m} \cdot \left( \partial _j \textbf{m} \times \partial _k \textbf{m} \right) , \end{aligned}$$

where (*i*, *j*, *k*) is an even permutation of (*x*, *y*, *z*). To detect a single Bloch point in a sample the integral can be computed over the whole sample surface and the exact position of the Bloch point does not need to be known.

**Figure 6:**
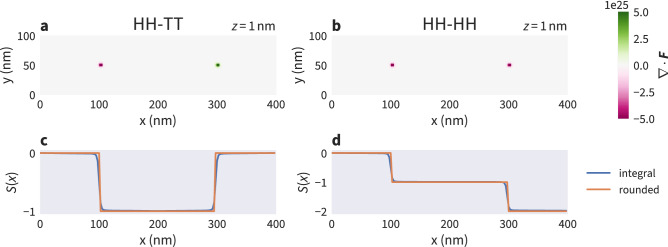
Classification of the two configurations containing two Bloch points shown in Fig. 2. The divergence of the emergent magnetic field for the two opposite- and same-type Bloch points is shown in panels (**a**) and (**b**), respectively. The *xy* plane visualised here is located at $z=1\,\textrm{nm}$, just above the interface. (**c**, **d**) The result of the convolution (5) which is used to identify the occurrence of Bloch points and their type due to the steps $\Delta S = \pm 1$.

This method is not directly applicable to multiple Bloch points when their positions are unknown: the sign of the topological charge of a Bloch point depends on its type (HH: $S=-1$, TT: $S=+1$). Therefore, contributions to the surface integral from Bloch points of opposite type cancel out. Figure 6 shows the divergence of the emergent field $\nabla \cdot \textbf{F}$ for a HH and a TT Bloch point (a) and two HH Bloch points (b), the two configurations discussed in Fig. 2.

To classify nanostrips that potentially contain multiple Bloch points we compute the convolution of the divergence of the emergent magnetic field with a Heaviside step function $\Theta$:

5 $$\begin{aligned} S(x) = \frac{1}{4\pi } \int _{V'}\textrm{d}^3r' \, \Theta \left( x - x'\right) \nabla _{\textbf{r}'} \cdot \textbf{F}\left( \textbf{r}'\right) . \end{aligned}$$

Due to numerical inaccuracies the result of the integral deviates from integer values. By translating the surface integral into a volume integral over the divergence of the emergent magnetic field using the divergence theorem the accuracy can be improved by roughly one order of magnitude.

In our set-up Bloch points are expected to be distributed along *x* following the strip geometry which justifies computing *S* as a function of *x*. This convolution can be interpreted as computing a series of integrals over increasing subvolumes $V'$ of the nanostrip starting at the left boundary ($x=0\,\textrm{nm}$). We round *S*(*x*) to integer values and count steps $\Delta S$ in this function.

Figure 6c and d show *S*(*x*) for the two example configurations. A head-to-head Bloch point is identified by $\Delta S = -1$, a tail-to-tail Bloch point by $\Delta S = +1$ corresponding to the topological charge of a Bloch point being $S = \pm 1$. Rounding to integer values is justified because deviations from integer values in the integral are a direct consequence of the limited accuracy due to the discretisation. The deviation from integer values decreases with decreasing cell size (see supplementary Fig. 3 for details).

## Supplementary Information

Supplementary Information.

## Data Availability

All results obtained in this work can be reproduced from the repository in Ref.^38^ which contains Jupyter notebooks^39^ to rerun the micromagnetic simulations and recreate all data and plots. In the repository pre-computed datasets are also available.
