# Supplementary material for: Bloch points in nanostrips
Source: Sci Rep. 2023 Apr 27;13:6910. doi: 10.1038/s41598-023-33998-z (PMC10140021; doi:10.1038/s41598-023-33998-z)
Supplement: Supplementary file 1 — Supplementary Information. [file 41598_2023_33998_MOESM1_ESM.pdf]

Bloch points in nanostrips – Supplementary material

Martin Lang, Marijan Beg, Ondrej Hovorka, and Hans Fangohr

I. INITIALISATION AND ENERGY MINIMISATION SCHEME

In the main text we have explained that we divide the nanostrip into multiple subregions to initialise the system and enforce the formation of a specific number of Bloch points during the energy minimisation. Here, we show two different examples for configurations containing one and two Bloch points, respectively.

Figure 1 shows the eight subregions that are used to obtain a configuration containing two Bloch points. Four subregions are located below $z = 0$ and have a negative DM energy constant D, and four above $z = 0$ with positive D. The four small subregions, shown with solid lines, are located at the top and bottom sample boundary. Each of the small subregions is contained within one surrounding subregions shown with dashed lines. The strip geometry in Fig. 1 is stretched in the z direction for better visibility.

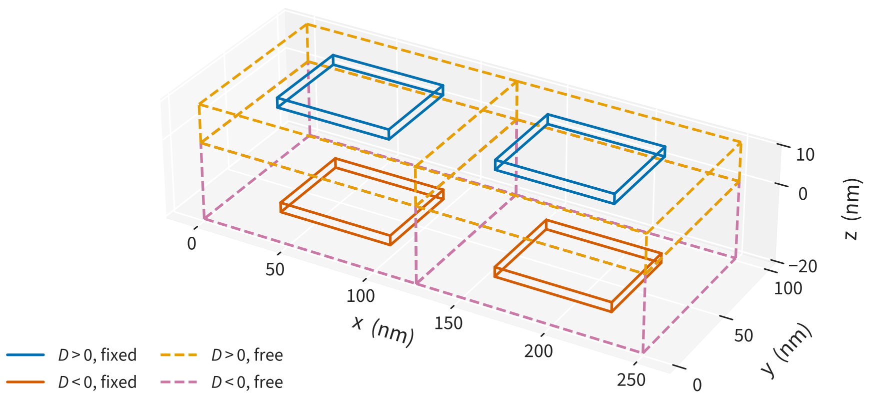

FIG. 1. Subregions used for the initialisation and fixed energy minimisation, and to define the two-layer geometry with opposite chirality (opposite sign of D) in the two layers for a nanostrip that shall contain two Bloch points. The magnetisation inside the subregions shown with solid lines is kept fixed during the first energy minimisation. Magnetisation in the top fixed subregions is initialised with reversed z component of the magnetisation (see Fig. 2). In the plot the z axis is stretched for better visibility. The fixed subregions are located at the strip boundary.

As outlined in the main text, the system initialisation and energy minimisation is done in three steps. The magnetisation after each step is show in Fig. 2 for a configuration containing a single HH Bloch point. The system geometry is $100\,\mathrm{nm} \times 100\,\mathrm{nm} \times (10 + 20)\,\mathrm{nm}$. This system only needs four subregions, *i.e.* one “column” of subregions in Fig. 1 (*e.g.* only the subregions for $x < 125\,\mathrm{nm}$).

During the initialisation step the magnetisation is initialised uniformly throughout the sample with $\mathbf{m} = (0, 0, 1)$. The only exception is the small subregion at the top sample boundary where the z component of the magnetisation is reversed, $\mathbf{m} = (0, 0, -1)$ (Fig. 2a). During the first energy minimisation the magnetisation in the small subregions at the sample boundary, shown with solid lines in Fig. 1 and highlighted in Fig. 2b, is fixed. This enforces the formation of the Bloch point in a controlled manner. The first energy minimisation leads to the magnetisation configuration shown in Fig. 2b. During the second energy minimisation, the magnetisation in all cells can freely change to allow the system find the local energy minimum, leading to the magnetisation shown in Fig. 2c.

II. CLASSIFICATION ACCURACY

In the methods section of the main text we have introduced our classification method. In short, to classify nanostrips that contain multiple Bloch points we compute the convolution of the divergence of the emergent magnetic field $\mathbf{F}$ with a Heaviside step function Θ:

$$S(x) = \frac{1}{4\pi} \int_{V'} \mathrm{d}^3 r' \, \Theta(x - x') \nabla_{\mathbf{r}'} \cdot \mathbf{F}(\mathbf{r}'). \tag{1}$$

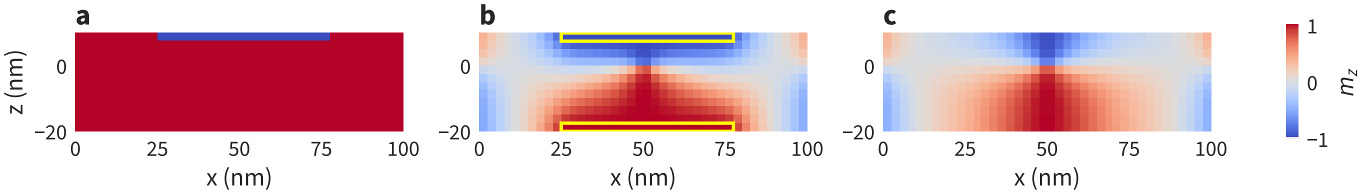

FIG. 2. Initialisation and energy minimsation for a single head-to-head Bloch point (cross-section at $y = 50\,\mathrm{nm}$). (a) The magnetisation is initialised with $\mathbf{m} = (0, 0, 1)$ with the exeption of a top subregion where $\mathbf{m} = (0, 0, -1)$. (b) During the first energy minimisation the magnetisation is kept fixed inside the yellow-highlighted subregions (see Fig. 1 for a 3D plot). The formation of a Bloch point is enforced by the opposite m_z values in the two fixed subregions. (c) During the second energy minimisation magnetisation in all cells can freely change.

The components of $\mathbf{F}$ are defined as:

$$F_i = \mathbf{m} \cdot (\partial_j \mathbf{m} \times \partial_k \mathbf{m}) , \tag{2}$$

where (i, j, k) is an even permutation of (x, y, z). The integral generally has non-integer values due to numerical inaccuracies resulting mainly from the finite discretisation cell size. To simplify the classification and counting we round $S(x)$ to integer values whereby we obtain sharp steps ($\Delta S = \pm 1$) at Bloch-point positions.

To justify the rounding to integer values we compare the deviations of $S(x)$ from integer values for different cell sizes. Figure 3a shows a configuration containing eight Bloch points in the pattern TT-HH-TT-TT-TT-TT-HH-TT. We compute $S(x)$ for three different cell sizes with cubic cells with edge lengths $l_\mathrm{c} = 5\,\mathrm{nm}$ (Fig. 3b), $l_\mathrm{c} = 2.5\,\mathrm{nm}$ (Fig. 3c), and $l_\mathrm{c} = 1\,\mathrm{nm}$ (Fig. 3d). For each cell size we show the integral result (solid lines) and rounded values (dashed lines). We can see that the difference (*i.e.* the inaccuracy) significantly decreases with decreasing cell size. For $l_\mathrm{c} = 1\,\mathrm{nm}$ integral and rounded values cannot be distinguished visually. All simulations in the paper are done with a cell edge length $l_\mathrm{c} = 2.5\,\mathrm{nm}$.

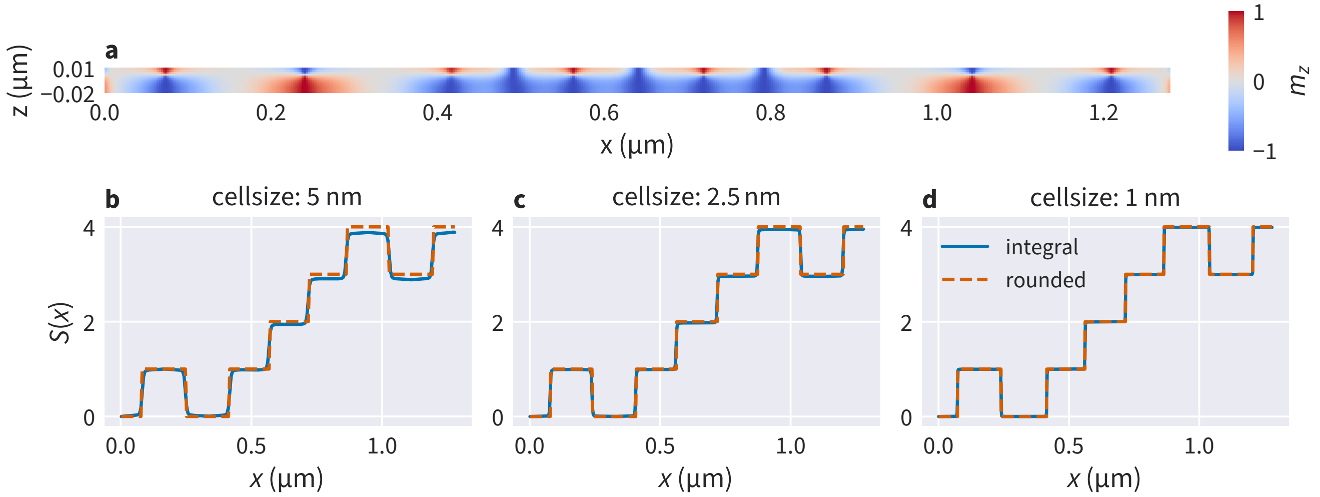

FIG. 3. Accuracy of the classification for a configuration containing eight Bloch points. (a) shows a cross-section of the magnetisation of configuration TT-HH-TT-TT-TT-TT-HH-TT in the xz plane located at the Bloch-point position ($y = 50\,\mathrm{nm}$). With decreasing cellsize – cell edge lengths: 5 nm (b), 2.5 nm (c), and 1 nm (d) – the difference between the integral $S(x)$ and the corresponding integer values decreases.
